# LEISH2b - A phase 2b study to assess the safety, efficacy, and immunogenicity of the Leishmania vaccine ChAd63-KH in post-kala azar dermal leishmaniasis

**DOI:** 10.12688/wellcomeopenres.17951.1

**Published:** 2022-08-03

**Authors:** Charles Lacey, Ahmed Musa, El Tahir Khalil, Brima Younis, Mohamed Osman, Rebecca Wiggins, Ada Keding, Paul Kaye

**Affiliations:** 1 York Biomedical Research Institute, University of York, UK, York, UK; 2 Institute of Endemic Diseases, University of Khartoum, Khartoum, Sudan; 3 York Trials Unit, University of York, UK, York, UK

**Keywords:** PKDL, Leishmania, Sudan, RCT

## Abstract

**Background:** The leishmaniases are neglected tropical diseases caused by various *Leishmania* parasite species transmitted by sand flies. They comprise a number of systemic and cutaneous syndromes including kala-azar (visceral leishmaniasis, VL), cutaneous leishmaniasis (CL), and post-kala-azar dermal leishmaniasis (PKDL). The leishmaniases cause significant mortality (estimated 20 - 50,000 deaths annually), morbidity, psychological sequelae, and healthcare and societal costs. Treatment modalities remain difficult. E.g., East African PKDL requires 20 days of intravenous therapy, and frequently relapsing VL is seen in the setting of HIV and immunodeficiency. We developed a new therapeutic vaccine, ChAd63-KH for VL / CL / PKDL and showed it to be safe and immunogenic in a phase 1 trial in the UK, and in a phase 2a trial in PKDL patients in Sudan.

**Methods: **This is a randomised double-blind placebo-controlled phase 2b trial to assess the therapeutic efficacy and safety of ChAd63-KH in patients with persistent PKDL in Sudan. 100 participants will be randomly assigned 1:1 to receive placebo or ChAd63-KH (7.5 x10 ^10^vp i.m.) at a single time point. Follow up is for 120 days after dosing and we will compare the clinical evolution of PKDL, as well as the humoral and cellular immune responses between the two arms.

**Discussion: **Successful development of a therapeutic vaccine for leishmaniasis would have wide-ranging direct and indirect healthcare benefits that could be realized rapidly. For PKDL patients, an effective therapeutic vaccination used alone   would have very significant clinical value, reducing the need for extensive hospitalization and chemotherapy. Combining vaccine with drug (immuno-chemotherapy) might significantly increase the effective life of new drugs, with lower dose / abbreviated regimens helping to limit the emergence of drug resistance. If therapeutic benefit of ChAd63-KH can be shown in PKDL further evaluation of the vaccine in other forms of leishmaniasis should be considered.

**Clinicaltrials.gov registration:** NCT03969134.

## Introduction

### The need for a therapeutic vaccine against VL / PKDL

Human visceral leishmaniasis (VL), also known as kala azar, is a neglected tropical disease. With 95% of cases occurring in India, Bangladesh, Nepal, Sudan and Brazil, VL preferentially occurs in the most impoverished ^1^. With an estimated 20,000 - 50,000 deaths annually, mostly children and young adults, VL ranks second only to malaria amongst parasitic infections for mortality, and as measured by disability-adjusted life years (DALYs) lost, it ranks in the top 10 infectious diseases globally. In some developing country regions, there is epidemiologic concurrence, and clinical interactions between VL and HIV, and in East Africa, co-infection rates of 34% have been reported ^2^. During VL and after treatment, post kala azar dermal leishmaniasis (PKDL) may develop. PKDL is a complex, chronic, and disfiguring skin disease that manifests as abundant nodular, papular, or hypo-pigmented macular skin lesions. Granulomas with few parasites, residual parasite antigen and mononuclear cell inflammation are the main histopathological features. PKDL significantly affects quality of life and is often mistaken for leprosy. PKDL patients are also reservoirs for onward VL transmission. In Sudan, PKDL occurs in ~30–60% of patients cured from VL. Spontaneous healing within six months is the rule, but in ~15% of patients, mostly children, lesions persist. Treatment for PKDL in Sudan requires hospitalisation (AmBisome ^®^, 50mg/kg/20 days), with significant costs for healthcare providers, patients, and households ^3– 6^. 

The potential emergence of resistance to existing anti-leishmanial drugs in East Africa is a significant threat, as is the apparent poor efficacy in East Africa of drugs currently effectively deployed in South-East Asia. Affordable second line treatments are often not available, and no single new therapies are in phase 2 or phase 3 trials, making the development of new preventative and/or therapeutic measures a major international research priority. World Health Assembly resolution EB118.R3 (Geneva 05/07) “calls on partner bodies… to accelerate research on, and development of, leishmaniasis vaccines” ^7^. 

A therapeutic vaccine would have wide-ranging direct and indirect healthcare benefits that could be realized rapidly (United Nations Children’s Fund (UNICEF) / United Nations Development Programme (UNDP) / World Bank / World Health Organization (WHO) special programme for research and training in Tropical Diseases (TDR), and the Infectious Diseases Research Institute (IDRI) (Seattle) Consultation Report ^3, 5^). For PKDL patients, an effective therapeutic vaccination used alone would have very significant clinical benefits, reducing the need for extensive hospitalization and chemotherapy. Combining vaccine with drug (immuno-chemotherapy) might significantly increase the effective life of new drugs, with lower dose / abbreviated regimens helping to limit the emergence of drug resistance. Finally, the capacity development accompanying therapeutic trials and their simplicity in design makes such trials an important stepping-stone in leishmaniasis elimination programmes. Prior human trials of combined immuno-chemotherapy of PKDL provided optimism that therapeutic vaccines against leishmaniasis can be developed ^3^. 

No effective vaccine has yet been developed for VL / PKDL despite significant research efforts. Unsuccessful prophylactic vaccine trials in humans have employed crude antigen mixtures, autoclaved parasites, or defined antigens chosen for their abundance and/or immunogenicity during natural infection and have adopted vaccination regimens designed to target CD4 ^+^ T cells. In contrast, and based on the importance of CD8 ^+^ T cells for protection against leishmaniasis and a range of other intracellular pathogens, we have sought to develop a novel therapeutic vaccine for VL / PKDL, biased towards the induction of CD8 ^+^ T cell responses.

### Rationale for a therapeutic CD8 ^+^ T cell-inducing vaccine against leishmaniasis

Our approach, of inducing and expanding host CD8 ^+^ T cell responses, is rooted in a long history of research. Our work, along with that of others, has demonstrated the importance of CD8 ^+^ T cells for primary resistance, resistance to re-challenge and vaccine induced resistance against experimental infection with *L. infantum* and *L. donovani*, the causative agents of VL / PKDL ^8^. Following prophylactic vaccination against experimental VL, CD8 ^+^ T cells are the major correlate of protection and, where tested, essential for efficacy. For example, the protection against *L. donovani* induced by both a kinetoplastid membrane protein-11 (KMP-11) -DNA vaccine and a hydrophilic acylated surface protein B1 (HASPB) protein vaccine was associated with strong antigen-specific CTL responses and IFN-γ generation ^9– 11^. Clinical studies also increasingly point to a host protective role for CD8 ^+^ T cells. The frequency of activated CD8 ^+^ T cells is substantially increased in asymptomatic and treated VL patients, compared to untreated controls, and *Leishmania* specific CD8 ^+^ T cells have been identified as an important contributor to anti-leishmanial immunity in asymptomatic VL patients ^12^. Direct evidence that a therapeutic vaccine targeting CD8 ^+^ T cells may be both beneficial and achievable in VL stems from three sets of observations. First, we have directly demonstrated therapeutic vaccination in experimental models of VL, dependent upon induction of CD8 ^+^ T cells ^13^. Second, using a model of adoptive cellular immunotherapy, we have shown that CTL, as well as central and effector memory CD8 ^+^ T cells can be re-activated in mice with ongoing VL, leading to reduced parasite burden ^14^.Third, the pathology associated with established experimental VL is similar to that observed in human disease. Recent studies confirm the similarity of regulatory T cell responses in human and murine VL ^15^ and by adoptive transfer, we have shown that in spite of disruption to the splenic microenvironment, the capacity to prime CD8 ^+^ T cells remains intact ^14^. Collectively, therefore, these data demonstrate that both priming of naïve CD8 ^+^ T cells and the activation of pre-existing effector/memory CD8 ^+^ T cell responses can occur in the face of disease-associated pathology and of pre-existing, IL-10-mediated immune regulation. When present at an appropriate frequency, antigen specific CD8 ^+^ T cells can thus provide therapeutic benefit. The challenge for therapeutic vaccination, therefore, is to similarly enhance CD8 ^+^ T cell frequencies in man, breaking or bypassing any pre-existing regulatory control of CD8 ^+^ T cell function. Viral vectors such as Adenovirus or MVA have been shown in several clinical settings to be the carriers of choice to induce CD8 ^+^ T cells ^16– 18^. Currently available viruses can accommodate more foreign DNA than the sequences encoding either or both vaccine antigens proposed here.

### HASPB and KMP11 (KH) as vaccine antigens, and the development of ChAd63-KH

We identified and selected two antigens (HASPB and KMP11) for which experimental protection data were most compelling and which were weakly if at all recognized by CD8 ^+^ T cells during experimental or human VL ^19^. HASPB is known to feature stretches of 11–14 amino acid-long repeats, and so to minimize any impact of isolate-specific variation in the repeat regions, we designed a synthetic HASPB gene comprising the conserved N and C termini flanking 10 repeats from the 17 repeats identified to date. These have been arranged to preserve their native order and re-iteration as observed in multiple Indian field isolates, and to maintain natural protein length. This unique gene was termed HASPB consensus. KMP11 is highly conserved. Both proteins were expressed from a single vector using the 2A sequence from TaV virus ( http://www.st-andrews.ac.uk/ryanlab/2A_2Alike.pdf). During translation, the polyprotein is processed at the 2A sequence resulting in each protein being expressed as a single cleavage product. 2A sequences have been approved for use in human gene therapy by the U.S Food and Drug Administration (FDA) ^20^ but had not previously been used in human vaccines. Our pre-clinical and clinical data indicates that this novel approach has been highly successful. 

To evaluate the efficacy of a pre-clinical adenoviral vectored polyprotein vaccine (termed here HuAd5-KH) as a stand-alone therapy, we evaluated parasite burden in the spleens of infected mice. The spleen is relatively refractory to drug treatment providing a severe therapeutic challenge, even for drugs established in the clinic. For example, administration of sodium stibogluconate (300mg/kg i.v) results in only 11–52% suppression of parasite burden and multiple doses at 8mg/kg of Ambisome (liposomal amphotericin B) cannot achieve greater than 80% efficacy ^21, 22^. Hence, we set our criteria for effective single dose therapeutic vaccination as a suppression of splenic parasite growth of > 50%. The therapeutic efficacy of 10 ^9 ^pfu HuAd5-KH administered as a single dose i.d. was 66 ± 8% (p=0.001 vs. unvaccinated mice), with a response rate of 91% (21/23 animals tested). HuAd5-KH thus constitutes an effective stand-alone therapeutic intervention in this experimental model of infection. Vaccination also induced potent CD8+ T cell responses against multiple epitopes within the KH polypeptides ^19^.

The prevalence of immunity to human adenovirus has prompted the development of simian adenoviruses as vectors for human clinical vaccine development. Simian adenoviruses exhibit hexon structures that are highly related to those of human adenoviruses. Indeed, Chimpanzee Adenovirus type 63 (ChAd63) hexons are most similar in sequence to HuAd4 hexons previously used by the US military in mass vaccination campaigns where over two million adults received serially passaged adenovirus and which showed good safety and efficacy data ^23^. Hexons are the major capsid proteins in adenoviruses; they are potently immunogenic and the main target of neutralising antibodies ^24^. In chimpanzee adenoviruses the E1 locus can be deleted to render viruses replication deficient and allow transcomplementation on an E1 HuAd5 complementing cell line ^25^. An additional attractive feature of the system is that the lack of sequence homology between HuAd5 and simian adenoviruses at the E1 flanking sequence prevents homologous recombination and production of replication competent virus. ChAd63 was first used in humans as a vaccine vector in 2007, and has since been administered to thousands of subjects with an excellent safety record.

Funded by the Wellcome Trust, we developed ChAd63-KH for human use, in collaboration with Okairos SRL. The Investigational Medical Product was manufactured to cGMP by Advent SRL, Pomezia, Italy (395 vials at 7.5x10 ^10^ vp/ml; vp/ifu ratio = 115; 0.65ml per vial). MHRA/REC approval for a UK first-in-human clinical trial was granted in early 2013 and we completed a successful first-in-human clinical trial of the new therapeutic vaccine for VL / PKDL, ChAd63-KH, in 2014 ^26^. This trial demonstrated the safety of ChAd63-KH in healthy UK adult volunteers and immunogenicity against the two vaccine-encoded *Leishmania* antigens on par with that seen to other vaccine candidate antigens in clinical development for other diseases (e.g., malaria, HCV, Ebola). Following external peer review of the data generated during LEISH1, a phase 2a study was funded in adults and adolescents with >six months PKDL. This trial successfully completed at the Dooka Centre for Tropical Medicine and the vaccine showed minimal adverse reactions in PKDL patients and induced potent innate and cell-mediated immune responses measured by whole-blood transcriptomics and ELISpot ^27^.

## Protocol

### Registration

This trial was registered on clinicaltrial.gov on May 31 ^st^ 2019; https://clinicaltrials.gov/ct2/show/NCT03969134. The trial protocol was developed following the SPIRIT guidelines ^28^.

### Study design

This is a randomised, double-blind, placebo-controlled phase 2b trial to assess the therapeutic efficacy and safety of ChAd63-KH in patients with persistent PKDL of > 6 months duration at a single centre in South-East Sudan. 100 participants will be randomly assigned (50 in each arm) to receive placebo or ChAd63-KH 7.5 x10 ^10^vp. Doses will be administered at a single time point. Follow up will be for 120 days after dosing and we will compare the clinical evolution of PKDL, as well as the humoral and cellular immune responses between the two arms.

### Study site

Professor El-Hassan’s Centre for Tropical Medicine, Dooka, Gedarif State, Sudan

### Objectives and Endpoints

***Primary Objective***

To assess the safety and efficacy of the *Leishmania* vaccine ChAd63-KH in patients with persistent PKDL. Volunteers aged between eight-50 years inclusive will be randomised to receive either the vaccine at 7.5 x10 ^10^vp, or placebo, with 50 participants in each arm. Doses will be administered at a single time point to patients under hospital care.

Safety will be measured by assessing adverse event data collected through history, clinical examination, blood tests and if necessary tissue specific parasitological confirmation.

Efficacy will be measured by the proportion of subjects at 42 and 90 days who do not need standard PKDL treatment as judged by the following criteria. The severity, extent and distribution of the PKDL disease will be measured by a standardized grading system, and image capture. If there is a <75% improvement in the degree of PKDL then the patient will be offered standard PKDL treatment of 20 days liposomal Amphotericin B. If there is a 75–90% improvement in the degree of PKDL then the CI in consultation with the patient will decide on conservative treatment or Amphotericin B. With >90% improvement then no further intervention will be recommended.

The grading system for PKDL is:

1. Scattered maculopapular or nodular lesions, covering mainly the face particularly around the mouth and eyes. 

2. Dense maculopapular or nodular rash covering most of the face and extending to chest, back, upper arms and legs

3. Dense maculopapular or nodular rash covering most of the body, including hands and feet. 

***Secondary Objectives***

1. To compare the humoral and cellular immune responses generated by the candidate vaccine in patients with persistent PKDL, compared with placebo-treated controls.

The specific immunological end points will be measures of T cell, B cell and innate immunity induced by the vaccine e.g. as measured by ELISPOT, flow cytometry, ELISA and/or transcriptomics. 

2. To observe any clinical changes in the cutaneous PKDL over a 120 day period following vaccination.

### Study procedures

***Number and Source of Volunteers***

We aim to recruit 100 patients with persistent PKDL for the study. Volunteers will be recruited through active case detection by the medical team visiting nearby clinics and villages known to be foci of endemic leishmaniasis in the areas around the study site. 

***Inclusion Criteria***

The volunteer must be:

•   Aged 12 to 50 years on the day of screening.

•   Females must be unmarried, single, or widowed.

•   Willing and able to give written informed consent.

•   For adolescents aged 12 to 17 years on the day of screening written informed consent must be obtained from a parent and assent must be obtained from the child.

***All Participants***

•   Uncomplicated PKDL of > six month’s duration.

•   Available for the duration of the study.

•   In otherwise good health as determined by medical history, physical examination, results of screening tests and the clinical judgment of a medically qualified Clinical Investigator.

•   Negative for malaria on blood smear.

•   Judged, in the opinion of a medically qualified Clinical Investigator, to be able and likely to comply with all study requirements as set out in the protocol. 

•   Willing to undergo screening for HIV, Hepatitis B and Hepatitis C.

•   Leishmania PCR positive on the screening skin biopsy.

•   For females only, willing to undergo urinary pregnancy tests on the day of screening, on the day of vaccination (prior to vaccination) and seven and 42 days after vaccination.

***Exclusion Criteria***

The volunteer may not enter the study if any of the following apply:

•   Has mucosal or conjunctival PKDL.

•   Has had treatment for PKDL within 21 days.

•   Negative for antibodies on RK39 strip test. 

•   Receipt of a live attenuated vaccine within 60 days or other vaccine within 14 days of screening. 

•   Administration of immunoglobulins and/or any blood products within the three months preceding the planned administration of the vaccine candidate.

•   History of allergic disease or reactions likely to be exacerbated by any component of the vaccine or a history of severe or multiple allergies to drugs or pharmaceutical agents.

•   Any history of severe local or general reaction to vaccination as defined as:

○ Local: extensive, indurated redness and swelling involving most of the antero-lateral thigh or the major circumference of the arm, not resolving within 72 hours.
○ General: fever ≥ 39.5°C within 48 hours, anaphylaxis, bronchospasm, laryngeal oedema, collapse, convulsions or encephalopathy within 48 hours.

•   Females – pregnancy, less than 12 weeks postpartum, lactating, or willingness/intention to become pregnant during the study and for three months following vaccination.

•   Seropositive for hepatitis B surface antigen (HBsAg) or Hepatitis C (antibodies to HCV).

•   Any clinically significant abnormal finding on screening biochemistry, haematology blood tests, or urinalysis.

•   Any confirmed or suspected immunosuppressive or immunodeficient state, including HIV infection; asplenia; recurrent, severe infections, and chronic (more than 14 days) immunosuppressant medication within the past six months.

•   Tuberculosis, leprosy, or malnutrition (malnutrition in adults defined as a BMI <18.5, and in children and adolescents (eight-17yrs) as a Z score cut-off value of <-2 SD).

•   Any other significant disease, disorder, or finding, which, in the opinion of a medically qualified Clinical Investigator, may either put the volunteer at risk because of participation in the study, or may influence the result of the study, or the volunteer’s ability to participate in the study.

•   Unlikely to comply with the study protocol.

***Screening, eligibility, and informed consent***

The following general eligibility questions will be discussed prior to conducting informed consent:

- Age.
- Availability for the duration of the study.
- General health state.
- Allergy status.
- History of Leishmaniasis infection.
- Contact details.

At the screening visit, the volunteer will be fully informed of all aspects of the trial, the potential risks, and their obligations. The Chief Investigator (or a study physician in accordance with the delegation log) has both an ethical and a legal responsibility to ensure that each volunteer being considered for inclusion in the study is given a full explanation of the study. A check of eligibility will be conducted and any questions about the study will be answered. If the volunteer is still willing and interested they will be asked to sign and date three copies of Consent Form 1 - one for the volunteer to keep, one to be stored in the volunteer’s case file, and one to be placed in the Trial Site File. 

To ensure informed consent, the following information will be provided by a member of the study team

- Pre-HIV test risk assessment and discussion.
- That it is unknown whether or not the study vaccine will cure them from their Leishmania infection, and that subsequent drug treatment will be given as required at the end of the vaccine follow up period (day 42 – day 90 post vaccination).
- The importance of continued follow up in the study to monitor any unforeseen events will be stressed.

After informed consent has been obtained, assessments and investigations will be undertaken according to the schedule. These include;

- Medical history.
- Vaccination history.
- General Examination.
- Collection of urine for urinalysis and, if female, a pregnancy test.
- Blood samples for routine laboratory investigations (haematology and biochemistry) and the blood borne viruses Hepatitis B, Hepatitis C and HIV.
- Skin biopsy from a typical PKDL lesion at a site acceptable to the patient.

Each volunteer who enters the trial by signing a copy of the consent form will be assigned an identification number. These numbers will not be reassigned. A screening log of screened volunteers with their identification number and demographic will be maintained and kept in the in the trial site file to track volunteers who have been screened for the study. If the volunteer is not enrolled into the dosing part of the trial, study staff will document the reason for not enrolling in the screening log.

A photograph will be taken for identification purposes and will be placed in the volunteer’s file only. Photographic records of lesions pre- and post- vaccination will also form part of the clinical file. Any photographs of the whole face will have the eyes blacked out.

Following the screening visit a medically qualified Clinical Investigator will review the results of each volunteer to determine if they are eligible for further involvement in the study. The Chief Investigator (or a medically qualified clinical investigator in accordance with the delegation log) must confirm eligibility for each volunteer based on the screening assessment. This must be documented in the case record form (CRF).

A medically qualified Clinical Investigator must confirm eligibility for each volunteer based on the screening procedures, including findings from clinical histories, examinations, laboratory results and clinician’s report. This must be documented in the CRF. If the volunteer is deemed to be eligible the dosing visit can be arranged.

If for any reason the volunteer is considered a screen failure the volunteer will be contacted by a Clinical Investigator, notified of all of their results and the reason for the screen failure. Volunteers failing screening will be treated with standard of care (SOC).

### Randomisation and study intervention

50 volunteers will receive a single intramuscular dose of ChAd63 KH 7.5x10 ^10^ vp (IMP) and 50 will receive a placebo injection (normal saline). The clinical investigators and the participant will be blinded as to which injection they receive. The IMP will be stored below -60°C. Prior to administration the IMP will be removed from the dry ice and allowed to defrost to room temperature. Within one hour of the IMP being removed from the freezer it will be administered to the volunteer. An assistant pharmacist and study nurse will prepare the vaccine / placebo injections. There will be stratification between patients aged eight-17 yrs, and patients aged 18–50 yrs, to ensure a balanced randomisation within those age strata. The pharmacist will be told which age strata the patient is in by a study nurse, and will prepare the injection with the study nurse but without the presence of any other study personnel. There will be two series of sealed envelopes, one for each of the adolescent and adult cohorts. Envelopes will be in order of a pre-determined randomisation list, with equal allocation to vaccine or placebo within each block (cohort) of 50. These two series of envelopes will have large clear easily distinguishable external labelling, with the cohort, and successive cohort patient numbering (e.g., YOUTH 1,2,3 …, or ADULT 1,2,3 …). Within each envelope is the randomisation to VACCINE or SALINE. The vaccine and placebo injections will be prepared in blacked out syringes labelled with the patient Identification Number (ID No.) according to a study standard operating procedure (SOP). The study nurse will then take the prepared injection to the clinical team who will administer the injection. The study nurse must take care not to communicate the randomisation to the clinical team, nor to note this in the CRF or other documentation. The syringe will be further labelled with the patient’s initials, and the date and time of injection, and after injection returned to the pharmacist for accountability and monitoring. The cohort number, and the date and time of administration will also be recorded in the CRF.

The dose volume will be injected into the deltoid muscle of the upper arm using a 21–23 gauge needle long enough to reach deep into the muscle. The needle will be inserted at an angle of approximately 90° to the skin. Volunteers receiving the vaccine will be asked which arm they would like to be injected. Full in-patient clinical care will be available during, and for at least 48 hrs after vaccination. 

### Study interventions

The schedules of visits are presented below in Table 1. Volunteers will be required to stay in hospital for three days and be monitored as an outpatient out to 120 days post vaccination. Time 0 will start on the day of vaccination. Volunteers with persisting PKDL sufficient to need treatment (see Primary Objective, above) between days 42–90 will be admitted to hospital for the Standard of Care (usually 20 days intravenous liposomal Amphotericin B). A final monitoring visit will be made at day 120 post vaccination. 

**Table 1.** Schedule of events.

	Screening	Vaccination ^d^	In patient monitoring	In patient monitoring	Outpatient Follow up	Outpatient Follow up	Outpatient Follow up	Outpatient D90	Outpatient D120
Visit / Observation Number	1	2	3	4	5	6	7	8	9
Timeline (Days)	-	0 *	1	3	7	21	42	90	120
Window	-28 to -4 days	-	+/-3 hours	+/- 1 days	+/- 1 days	+/- 3 days	+/- 3 days	+/- 10 days	+/- 15 days
Vaccination		X							
Medical History/Adverse event reporting	X	X	X	X	X	X	X	X	X
General Examination, vital signs	X	X ^a^	X ^a^	X ^a^	X ^a^	X ^a^	X ^a^	X	X
Height and weight	X							X	X
Examination of administration site		X ^b^	X	X	X				
PKDL examination, grading & recording	X	X	X	X	X	X	X	X	X
Skin biopsy	X ^c^							X ^e^	
Urinalysis	X	X					X	X	X
Urinary pregnancy test (females only)	X	X ^d^			X		X		
Haematology & Biochemistry (5ml)	X	X ^d^	X		X			X	X
Blood Borne Virus Screen(2.5ml)	X						X		
Bio-Rad LEISH IT (Leishmania antibody test) (1ml)	X								
Microarray bloods (2.5ml)		X ^f^	X	X	X				
Cellular Responses (10ml)		X				X (15ml)	X	X	X
Serum (2.5ml)		X					X		
Malaria RDT (0.5ml)	X					X	X	X	X
Blood Volume Per Visit	9.0ml	20.0ml (15ml in 8-11yrs)	7.5ml	2.5ml	7.5ml	15.5ml	15.5ml	15.5ml	15.5ml

* admission to hospital day before vaccination day ^a ^Full general examination only if required ^b ^At vaccination, examination of administration site and recording of observations will be done at 10 minutes, 60 minutes and 120 minutes post vaccination. ^c ^No skin biopsy at entry in 8-11 yr olds ^d. ^Repeat haematology and biochemistry, as well as repeat urine pregnancy test if female, do not need to be performed **if vaccination is within 48 hours** of the initial or repeat screening tests, or in 8-11 yr olds. ^e^ Inpatients receiving SOC treatment as an inpatient ^f^ Microarray blood sampling will be performed pre-vaccination.

***Visit 1: Screening and Enrolment***

All potential volunteers will have a screening visit, which can take place between one to 28 days before the vaccination. Informed consent will be undertaken at the screening visit before any screening procedures. The screening procedures will be undertaken as indicated in Table 1. Volunteers will be considered enrolled once informed consent has been taken.

If participants test positive for malaria at screening, then they will be treated with SOC and can be re-screened after seven days.

***Visit 2: Vaccination***

The eligibility of the volunteer will be reviewed following the screening assessment once all of the results from the screening visit have been considered. If eligible, a day 0 visit will be scheduled for the volunteer to receive the vaccination. A further assessment of eligibility will be conducted on the day of vaccination prior to the volunteer receiving the vaccine. Repeat blood tests (and pregnancy tests for females) are required if the vaccination visit takes place more than 48 hours after the initial screening visit or repeat screening visit. The importance of continuing to attend follow up visits after receipt of the vaccine to monitor any unforeseen events will again be stressed to the volunteer. Blood will also be taken for exploratory immunology analysis as detailed in Table 2.0. 

The vaccination will be into the deltoid muscle of an arm of the volunteers choosing. Following vaccination, the area of skin injected will be covered with a small light dressing and this will be removed after at least one hour. The dressing will be disposed of in line with local recommendations. 

All volunteers will be issued with a study volunteer identity card and encouraged to contact the research team if there are any problems. 

Volunteers will be required to stay in hospital for the first three days post vaccination. During this time, observations will be performed at 30 minutes and two hours, and then at 24 hrs and three days after vaccination.

***Outpatient Follow Up Visits***

Further visits will take place at seven, 21, 42, 90, and 120 days after vaccination. These will be compliant with the protocol if they take place +/-3 days either side of the target date (D21 and D42) or +/- 10 days (D90) or +/- 15 days D120) determined by the date of vaccination.

Volunteers will be assessed for local and systemic adverse events using a focused history, physical examination and malaria blood tests. Blood will also be taken for exploratory immunology analysis as detailed in Table 1.

### Study procedures

Procedures will be performed on the visit time points indicated in Table 1. Additional procedures or laboratory tests may be performed, at the discretion of a Clinical Investigator if clinically required.

Observations which will be documented are blood pressure, pulse rate and temperature

Blood tests comprise the following laboratory tests:

- Haematology: Haemoglobin, White blood cells, Neutrophils, Lymphocytes, Platelets.
- Biochemistry: Creatinine, ALT, AST, Albumin, Total Bilirubin, Direct Bilirubin
- Blood Borne Virus Screen: Hepatitis B surface antigen, HIV antibodies, Hepatitis C antibodies.
- Bio-Rad IT LEISH rapid test (RK39 Leishmania antibody test) will be conducted at the trial site.
- Immunology: KMP HASPB antibodies, microarray bloods and cellular responses.
- RDT for Malaria parasites. If positive appropriate treatment will be given.

Dipstick for urinalysis will be conducted at the trial site for the presence of leucocytes, protein, and blood. In female volunteer’s urine will be tested for beta-human chorionic gonadotrophic (HCG) at screening, at the vaccination visit (prior to vaccination), at visit five, and at the final study visit.

General examination will include cardiovascular, respiratory, neurological, inspection of administration site, and palpation of axillary and cervical lymph nodes. Abdominal examination with emphasis on spleen and liver size below costal margins at screening and follow up visits will be performed and recorded. Height and weight will be measured at the screening visit and at visit eight.

Skin biopsy will be performed at screening in all subjects from what appears to be a typical PKDL lesion selected by the investigator at a (usually inconspicuous) site agreeable to the patient. Biopsy is under local anaesthesia with a 4mm punch. The sample is divided into two cryotubes, one into a stabilising solution for PCR, and one into formalin for immunohistology. In non-responders / subjects having SOC treatment a further punch biopsy will be taken from what appears to be a persistent PKDL lesion, at a site chosen as above, and processed, tested and stored as above.

### Management of biological samples

Blood samples taken at the screening visit for routine laboratory parameters and blood borne viruses as well as routine safety parameters during the trial will be tested at the Dooka clinical laboratory. Blood samples for immunogenicity testing will be analysed at the Institute for Endemic Diseases, University of Khartoum. Whole blood for transcriptomics will analysed at the University of Siena. These samples will be identified by the volunteer’s trial number, date of birth, and initials as required the laboratory.

### Sample size calculation

This is a safety and efficacy study of a *Leishmania* vaccine. We estimate that a ≥25% reduction in patients requiring chemotherapy would have clinical utility. A total sample size of 100 (50 volunteers will be vaccinated with ChAd63-KH and 50 with placebo) would be sufficient to detect a significant difference between randomised groups, assuming 90% power, 5% statistical significance, a spontaneous clearance rate of ≤2% and loss to follow-up of in randomised patients of ≤5%. The number of participants is typical for early phase vaccine studies.

### Statistical and analytical plans

All patient characteristics and data collected at baseline and follow-up will be presented descriptively by age group and trial arm at each available time point as well as the change between time points where appropriate. Continuous measures will be reported as averages (n, mean, standard deviations, median, IQR, min, max) while categorical data will be reported as counts and percentages. Particularly skewed data or outliers will be highlighted. The flow of patients through the trial will be illustrated with a CONSORT diagram and reasons for missing data outlined.

PKDL based clearance rates (requiring no further treatment) will be compared between treatment arms using logistic regression at day 42 and day 90, adjusting for covariates including age group. Relative risk ratios will be presented with 95% confidence intervals and associated p-values. Recovery will be further illustrated by presenting the extent and grading of PKDL lesions as well as median percentage improvement by group at all available follow-up points.

The number of local and systemic adverse events will be tabulated by type and trial arm. The median number of adverse events per participant (separately for local and systemic events) will be compared between groups using the Mann-Whitney U test. Immunological end points will be compared between arms. The study statistician will not be blind to treatment allocation. However, the analyses will be checked by a further independent statistician before the release of results.

### Data collection and management

Primary data is collected at site in CRFs. All consenting volunteers will be allocated a CRF, although the hospital notes will also act as source data. The CRF will hold personal identifiable information on the volunteer, including name, address, date of birth, and volunteer trial number. The volunteer’s file will be held at the clinical site in a secure location. Permission will be obtained as part of the informed consent process to allow the research team and other responsible individuals access to the volunteers’ trial records.

Data collected directly from the volunteer or from medical examinations will be entered directly into the CRF. All laboratory reports will be filed in the CRF after review and sign off by a medically qualified Clinical Investigator. Data collected at the clinical site will be transcribed directly onto case report forms. The type of data to be recorded in the CRF will be in line with the details provided in the study schedule section. Appendix six of the protocol defines the source data in this trial ^28^. CRF’s will be identified with volunteer trial number and initials. No personal identifiable information will be sent outside of the hospital. 

The Clinical Research Organization (CRO) ClinServ International will undertake data management responsibilities as delegated by the University of York, which include the provision of study database, data entry and validation procedures. The CRO will work with the trial team to draft the CRFs. Local appropriately trained staff (named on a delegation log) will complete and send CRFs to the CRO for entry into the study database.

A screening log containing hospital numbers, name, date of birth, and whether the volunteer was enrolled into the dosing part of the study will be kept in the trial site file, which will be kept in a secure location at the trial site, with access restricted to study staff and monitors only.

A volunteer log containing hospital numbers, trial number, name, date of birth and whether the volunteer was enrolled into the dosing part of the study will be kept in the trial site file which will be kept in a secure location at the trial site, with access restricted to study staff and monitors only.

Volunteers will be identified only by their trial volunteer number, initials and date of birth on any documentation or samples that leave the trial site. No personal identifiable data will be stored with external organisations. Volunteers will not be identifiable in any study report or publication.

### Data preservation, sharing, and access

The investigators will maintain appropriate medical and research records for this trial. The Chief Investigator, co-investigators and clinical research nurses will have access to records. The investigators will permit authorised representatives of the sponsor, and regulatory agencies to examine (and when required by applicable law, to copy) clinical records for the purposes of quality assurance reviews, audits and evaluation of the study safety and progress. The study protocol, documentation, data and all other information generated will be held in strict confidence. No information concerning the study or the data will be released to any unauthorised third party, without prior written approval of the sponsor.

### Safety reporting

The guidelines for safety reporting within this clinical trial are based on the International Conference on Harmonization principles of good clinical practice (ICH GCP). Adverse events (AEs) will be classified and graded according to the Common Terminology Criteria for Adverse Events (CTCAE) system. All Serious and grade three or four AEs will be compared between arms and reported by frequency per arm. A Data and Safety Monitoring Board (DMSB) comprised of independent experts will be appointed and oversee the safety of the trial participants. All grade three or four, or serious AEs and adverse drug reactions including suspected unexpected serious adverse reactions (SUSARs), whether expected or not, will be recorded in the CRF.

***Reporting Procedures for Serious Adverse Events and SUSARs***

The study physician after discussion with the Chief investigator/Principal Investigator will complete an SAE report form and notify ClinServ, the Sponsor, the University of Khartoum Ethics Committee and the NMPB immediately (within 24 hours) of becoming aware of an SAE or SUSAR according to the ClinServ Adverse Events Reporting SOP. ClinServ and the Sponsor will assess such an adverse event, seek further clarification from the clinical investigators if necessary, and a full report will be sent to all the above named parties within seven working days of becoming aware of the event.

### Trial management

The Sponsor delegates certain roles and responsibilities to the Chief Investigator (CI)and the CRO, as detailed in the SOP entitled “Delegation of Tasks for LEISH 2b”.

A Clinical Trial Steering Committee (CTSC) will be formed comprising the CI, other Clinical Investigators based at the study site, an external independent expert and members nominated by the Wellcome Trust. The CTSC will be responsible for monitoring the progress of the trial and approving the protocol and any changes. It will meet at least once every three months during the course of the trial. Records of these meeting will be maintained. The CTSC will provide feedback to the DSMB.

A Trial Management Group (TMG) will be formed comprising the CI, other Clinical Investigators based at the study site, the UoY Clinical Investigator, the Project Lead, the CRO, with others co-opted as necessary. The TMG will meet by teleconference every month, unless a CTSC is scheduled. 

### Risk assessment

A full trial risk assessment will be carried out before commencement of the trial in accordance with the Sponsor’s SOP. 

### Monitoring

The conduct of the trial will be monitored by the CRO. Monitoring will be undertaken at regular intervals by suitably qualified and trained personnel in accordance with the SOP entitled ‘Monitoring of LEISH 2b’. The monitoring plan will be approved by the CTSC.

### Ethical and regulatory considerations

The Investigators will ensure that this study is conducted according to the principles of the current revision of the Declaration of Helsinki 2008 and in full conformity with the ICH guidelines for GCP (CPMP/ICH/135/95) July 1996.

***Research Ethics Committees***

A copy of the protocol, informed consent forms, any other written volunteer information, and the advertising material was submitted to and approved by the University of Khartoum (reference: FM/DO/EC, 31-03-2019) and the University of York Ethics Committees (reference: PK201910, 06-12-2019). Regulatory approval was granted by the National Medicines and Poisons Board, Sudan (reference: 0054596, 18-08-2019).

***Volunteer Confidentiality***

No material will be kept on file that refers to the study volunteer by their full name other than in source documentation kept at the trial site. The confidentiality of volunteers will be respected and maintained at all times. The volunteer’s trial number only will identify study reports. CRFs, associated database records and blood samples will only be identified by the volunteer’s trial number and date of birth.

***Risks***

The Investigators will ensure that the dignity, rights, safety and well-being of volunteers are given priority at all times. A risk assessment has been performed to assess the risks and benefits of trial participation to the individual volunteer safety, as well as the risks that underlie the validity of the trial results with respect to safety and immunogenicity outcome measurements. The outcome of this assessment has been used to guide the development of procedures with respect to informed consent, confidentiality, trial monitoring and audit. A genetically modified organism (GMO) risk assessment has also been undertaken and reviewed by the GMO safety committee at the University of York. This has informed the development of procedures for handling, storing and disposing of the vaccine.

### Expenses and benefits

Volunteers will be compensated for their travel and time and for the inconvenience caused by procedures at all study visits. Treatment of PKDL will be provided free of charge for those who need it. 

### Finance and Insurance

The study is funded by a Translation Award from the Wellcome Trust (WT108518). The University of York is the Sponsor for the project. The University of York has obtained a no-fault compensation policy for the study. This policy allows for compensation to participants in the study in the event of harm, without having to prove that the sponsor is at fault, where it is likely that such injury results from receiving the study vaccine or any other procedure carried out in accordance with this protocol for the study. The no fault compensation policy will indemnify employees of the Institute of Endemic Diseases against claims brought by study participants for injury, where conducting the study in accordance with this protocol.

### Publication policy

The results of the study will be analysed and prepared in a study report for publication in a peer reviewed professional journal. The Chief Investigator, Principal Investigator, Professor Charles Lacey, Professor Paul Kaye and the Statistical Investigator, Ada Keding, will form the basis of the writing group. Authorship will reflect work done by the Investigators.

### Archiving

All study documents will be securely stored at the University of York for a minimum of 15 years after the close of the trial in accordance with the sponsor’s SOP.

## Discussion

In this study, we will conduct a randomised double blind controlled trial of ChAd63-KH as a stand-alone therapeutic intervention for patients with persistent (>six months) PKDL in Sudan. We seek to provide evidence that single dose vaccination with ChAd63-KH will result in clinical cure of PKDL in at least 25% of patients within a 90-day timeframe. At this level of treatment success, single dose vaccination could make a significant difference to the clinical management and costs associated with this difficult to treat patient group. Current treatment for persistent PKDL cases in Sudan is based on liposomal amphotericin B (AmBisome ^®^, 2.5 mg/kg/day intravenously for 20 days) requiring hospitalisation and with a risk of nephrotoxicity and hypokalemia ^29^. Median direct costs for delivery of this treatment are estimated at over $350 ^30^. As PKDL is not a life-threatening disease, many patients are unwilling or unable (due to occupational or economic constraints) to undergo treatment, despite the stigma associated with the disease ^31^. Individuals with persistent PKDL maintain parasites in their skin for several months if not years and studies using xenodiagnoses have shown that PKDL patients can be a reservoir for *Leishmania donovani* transmission to sand flies ^32^. Hence, PKDL cases within the community can fuel the transmission of VL, a disease that is fatal if not treated. Therefore, interventions such as therapeutic vaccination that could reduce the severity or duration of PKDL may be expected to have a long-term impact on the incidence of VL. 

PKDL currently affects approximately 20% of patients in Sudan that receive the WHO recommended treatment for VL in Sudan (17 days of paromomycin / sodium stibogluconate). Of these, only approximately 20% develop persistent PKDL that lasts greater than 6 months. It is possible that infectiousness to sand flies varies between different PKDL phenotypes ^32^ but it is unclear whether the duration of an individual’s disease affects infectiousness parameters. Although this study has focused on persistent cases, which represent the highest bar to overcome for a therapeutic vaccine, if the study was successful it would pave the way to expand studies into the broader population of PKDL patients. Future studies might also seek to directly determine the impact of vaccination on infectiousness to sand flies. 

Recent clinical trials conducted by the Drugs for Neglected Diseases Initiative have evaluated new combination therapies for the treatment of VL, notably miltefosine plus paromomycin, and results are expected shortly, and other new medicinal products are soon to enter clinical trials in East Africa for the treatment of VL. It is hoped that these new drugs and combination therapies will prove to be more effective in the treatment of VL, have fewer side effects and also reduce the incidence of PKDL. However, opportunities are likely to remain for assessing the value of vaccination at the end of treatment for VL as a further means to reduce the incidence of PKDL (i.e., the development of a PKDL preventative vaccine). Modelling studies have demonstrated that at least for South Asia, administration of a PKDL-preventative vaccine would significantly add to the sustainability of VL elimination efforts ^33^.

Outside of East Africa, PKDL is restricted to South Asia, where there are significant concerns that PKDL patients may undermine elimination efforts against VL ^34^. PKDL in South Asia has distinct characteristics compared to that seen in Sudan, with both macular and polymorphic cases (the latter showing a combination of macular, nodular, and popular lesions). In South Asia, treatment with AmBisome ^®^ also appears less effective in clearance of cutaneous parasites ^35^, a result that may reflect the more chronic nature of the disease compared to that seen in Sudan. Whilst some of these distinctions between South Asian and African PKDL may result from differences in immune response, differences in parasite genotype cannot be excluded at this time. Hence, irrespective of outcome of the present study in Sudan, there would be value in evaluating the potential of ChAd63-KH in the context of PKDL in South Asia, particularly in the context of the current elimination campaign.

In addition to these clinical outcomes, we will also conduct in depth immune profiling using whole blood transcriptomics to evaluate vaccine-induced immunity and to determine whether there are biomarkers in blood that reflect either responsiveness to vaccine-induced cure or that predict self-cure. In our previously reported Phase 2a safety and immunogenicity study involving 24 patients ^27^, we observed that immune modules associated with antigen presentation and macrophage function were significantly over-represented in patients that successful cleared their PKDL within 90 -120 days of vaccination. However, this study did not have a placebo control and so we were not able to determine whether these immune response differences were related to vaccine response or reflected patient heterogeneity. The study also had insufficient power to stratify patients into those that were more likely to respond to vaccination or self-cure. These limitations will be largely overcome in the current study by comparison of whole blood transcriptomes pre- and post-vaccination across the placebo and vaccine arms. Identifying immune transcriptional biomarkers associated with self-cure and or vaccine-induced cure could lead to the development of rapid molecular tests that would enable stratification to treatment option or real-time analysis of treatment response. Similar approaches to guide patient management have been proposed recently for HIV/VL patients ^36^.

In conclusion, this study extends previous attempts using first- and second- generation vaccines and immunotherapies to develop an immune-based treatment for PKDL. ChAd63-KH is a third-generation vaccine employing a replication-deficient adenovirus for gene delivery into mammalian cells. The successful deployment of adenoviral vaccines during the COVID-19 pandemic has provided a broad global infrastructure able to support adenoviral vaccine manufacture and distribution ^37^. Should ChAd63-KH prove to be effective in the current study, this infrastructure may help to overcome many of the previous challenges associated with the development of leishmaniasis vaccines.

## Study Status

At the time of writing (02/08/22), 86 participants have been enrolled in the study and 68 participants have completed their follow up.

## Data availability

### Underlying data

No data are associated with this article.

### Extended data

Open Science Framework. LEISH2b - A phase IIb study to assess the safety, efficacy and immunogenicity of the Leishmania vaccine ChAd63-KH in post-kala azar dermal leishmaniasis. https://doi.org/10.17605/OSF.IO/EVKP3 ^28^.

This project contains the following extended data:

- LEISH2b protocol v0.92 270619.pdf. (Protocol information document).
- LEISH2b_ICF_May_2019 V1.0.pdf. (Information for patients about the study and informed consent form to be used).
- LEISH2b DSMB charter V1.0.pdf. (Data and Safety Monitoring Board charter).

### Reporting guidelines

Open Science Framework: SPIRIT checklist for ‘LEISH2b - A phase IIb study to assess the safety, efficacy and immunogenicity of the Leishmania vaccine ChAd63-KH in post-kala azar dermal leishmaniasis’. https://doi.org/10.17605/OSF.IO/EVKP3 ^28^.
