# Supplementary material for: LEISH2b - A phase 2b study to assess the safety, efficacy, and immunogenicity of the Leishmania vaccine ChAd63-KH in post-kala azar dermal leishmaniasis
Source: Wellcome Open Res. 2022 Aug 3;7:200. [Version 1] doi: 10.12688/wellcomeopenres.17951.1 (PMC10213822; doi:10.12688/wellcomeopenres.17951.1)
Supplement: Peer review history

# Peer review: Reviewer response for version 1

**DOI:** 10.21956/wellcomeopenres.19894.r57184

**Published:** 2023-05-25

**Authors:** Rakesh K Singh

**Affiliations:** 1 Department of Biochemistry, Banaras Hindu University, Varanasi, Uttar Pradesh, India

The study is aimed to evaluate the protective efficacy and safety of ChAd63-KH vaccine in subjects suffering from PKDL. The pre and post vaccination proceedings and protocols, starting from D0 to D120, are nicely presented and discussed. However, I would like to make a suggestion as I could not understand the purpose of measuring cellular responses at D42, 90, and 120 post vaccination. The measurements of cellular immune responses at the early days i.e., D7 and D14 (and D21) instead of D42 and D90 may provide some interesting information. This may impart an insight of CMI or humoral immune response at early days against vaccine antigens and their subsequent impact on T/B cells proliferation, function and memory. The measurements at D120 i.e., the final follow-up day is fine. This is just a suggestion, and thus it does not impact, in any way, the feasibility of the study and the protocols.

Is the study design appropriate for the research question?

Yes

Is the rationale for, and objectives of, the study clearly described?

Yes

Are sufficient details of the methods provided to allow replication by others?

Yes

Are the datasets clearly presented in a useable and accessible format?

Yes

Reviewer Expertise:

Visceral leishmaniasis (immunobiology and vaccines)

I confirm that I have read this submission and believe that I have an appropriate level of expertise to confirm that it is of an acceptable scientific standard.

# Peer review: Reviewer response for version 1

**DOI:** 10.21956/wellcomeopenres.19894.r51833

**Published:** 2022-08-18

**Authors:** Helen McShane

**Affiliations:** 1 Nuffield Department of Medicine, University of Oxford, Oxford, UK

This is a clearly written clinical trial protocol from an experienced team of investigators. I note the co-primary endpoint of safety and efficacy and perhaps this should be more clearly specified as a co-primary endpoint.

- One comment is under the Primary objective section the age range is 8-50 whereas later it is 12-50.
- One other comment is that the clinical follow-up should be blind to study group allocation and again this should be specified.
- The other comment is what response rate is expected in the placebo group - this information should be included in the stats section.
- What level of efficacy (75-90 or >90%) is the primary efficacy evaluation based on? This also needs specifying in the SAP. 

Is the study design appropriate for the research question?

Yes

Is the rationale for, and objectives of, the study clearly described?

Yes

Are sufficient details of the methods provided to allow replication by others?

Yes

Are the datasets clearly presented in a useable and accessible format?

Yes

Reviewer Expertise:

TB vaccine R&D, I have led many TB vaccine clinical trials including 2 efficacy trials.

I confirm that I have read this submission and believe that I have an appropriate level of expertise to confirm that it is of an acceptable scientific standard.
